# Spatial distribution of multielements including lanthanides in sediments of Iron Gate I Reservoir in the Danube River

**DOI:** 10.1007/s11356-021-13752-6

**Published:** 2021-04-14

**Authors:** Otilia Ana Culicov, Tatjana Trtić-Petrović, Roman Balvanović, Anđelka Petković, Slavica Ražić

**Affiliations:** 1 Frank Laboratory of Neutron Physics, Joint Institute for Nuclear Research, Dubna, Russian Federation; 2 National Institute for R&D in Electrical Engineering ICPE-CA, Bucharest, Romania; 3 Laboratory of Physics, Vinča Institute of Nuclear Sciences, National Institute of the Republic of Serbia, University of Belgrade, P.O. Box 522, Belgrade, 11001 Serbia; 4 “JaroslavČerni” Institute for the Development of Water Resources, Belgrade, Serbia; 5 Faculty of Pharmacy - Department of Analytical Chemistry, University of Belgrade, Belgrade, Serbia

**Keywords:** Instrumental neutron activation analysis (INAA), Iron Gate, Lanthanides, Multivariate analysis, Pollutants, River Danube, Sediments

## Abstract

Recent studies show that lanthanides (Ln) are becoming emerging pollutants due to their wide application in new technologies, but their environmental fate, transport, and possible accumulation are still relatively unknown. This study aims to determine major and trace elements including Ln in the Danube River sediment which either belong or close to the Iron Gate Reservoir. The Iron Gate Reservoir is characterized by accumulation of sediments as an effect of building hydropower dam Iron Gate I. The surface sediments were collected on the Danube River—1141 to 864 km and three tributaries along this waterway. Two samples of deep sediments were used for comparison. The results indicate the significant upward enrichment of Zn, Sb, Cr, Nd, and Dy in sediments belongs to the Iron Gate Reservoir. The sample 4-Smed is labelled as a hot spot of contamination with Zn, Cr, As, Sb, Nd, and Dy. Also, a trend of increasing concentration in the time period from 1995 to 2016 was found for elements Zn, Cr, and Ni in sediment samples in the Iron Gate Reservoir. Chemometric analysis shows the grouping of sample sites into clusters characterized by the following properties: (i) increased concentration of all measured elements (samples within the Iron Gate Reservoir); (ii) increased Cu concentration (11-Pek); and (iii) lower concentrations of the measured elements (deep sediments). The data presented hereby contribute to the monitoring of pollution of the River Danube sediments and give the first view of Ln profile in the studied sediments.

**Supplementary Information:**

The online version contains supplementary material available at 10.1007/s11356-021-13752-6.

## Introduction

Lanthanides (Ln) belong to the group of emerging contaminants with certain aspect of their potentially negative effect in the environment (Gwenzi et al. 2018). Also, Ln are a part of technology critical elements (TCE), e.g. elements with a high supply risk and economic importance, which rapidly increased in the last three decades (Massari and Ruberti 2013). TCE have been applied in high technology such as production of electronic devices (e.g. cell phones); in the energy production (hybrid vehicles, wind turbines); in the energy reduction (UV filters in glass, catalyst in chemical processes, and catalytic converter); in the energy efficiency (rechargeable batteries, energy-efficient lighting), as a contrast agent in medical imaging (magnetic resonance imaging, radiopharmaceuticals); and in the development of advanced weapon systems, etc. (Balaram 2019; Karn 2011). Also, TCE have been used in traditional industries such as petroleum refining, agriculture, metallurgy, and nuclear processing. According to UN Environment Programme (UNEP 2013), future economic development will be oriented towards renewable energy technologies, low carbon public transportation, clean energy vehicles, etc.; so it is directly dependent on various metals including TCEs. It should be stressed, the permitted concentration of TCEs in the environment is not regulated and monitored, they are trace elements which are detectable with analytical equipment with very low detection limits, and their possible human and environmental toxicity is not clear.

Like many heavy metals, Ln are normal constituents of minerals, and their concentration in the river sediments is related to surrounding geological source (Khan et al. 2017). Under natural conditions, Ln may only become available in small amounts via the groundwater and the atmosphere. Recently, Ln, as well as other TCEs, have been detected in various environmental compartments and influenced on environmental pollution and human health as a consequence of their increased industrial applications (Gwenzi et al. 2018). The sources of increasing concentration of Ln in the environment are domestic, industrial and mine wastewater, e-waste, recycling emissions, surface runoff, and atmospheric deposition (Nuss and Blengini 2018). In 1996, Bau and Dulski (1996) reported for the first time anthropogenic-originated gadolinium in river water from contrast agents for magnetic resonance imaging. Increasing concentrations of Sm and La were detected in the Rhine River derived from the industrial effluent from a facility producing a catalyst for petroleum refining (Kulaksiz and Bau 2007). Increasing concentrations of Ln were found in agricultural soils and human samples in the area of Ln mining and smelting (Meryem et al. 2016), as well as in sediments (Brito et al. 2018; Alkan et al. 2020). Because of the appearance of Ln and the platinum group of elements in higher concentration in the environment compared with their background, these elements are grouped into the fourth group of trace elements that are potentially toxic even at very low concentration and about which not much information is available (Balaram 2016; Bott 1995). Although, technological application and research interest for Ln have enhanced, environmental transport and fate, and possible accumulation of these elements and their impact on nature and human health, are still limited (Gwenzi et al. 2018; Vukojević et al. 2019; Técher et al. 2020). Regardless if quantification of TCEs is not covered by the EU monitoring programmes, the huge industrial applications of TCEs today and their appearance in different environmental compartments can lead to TCEs becoming future candidates for monitoring.

According to the European Union Water Framework Directive, sediments are one of the three major sources of river pollution (European Commission 2010; Directive 2013). Concentrations of the organic and inorganic pollutants adsorbed on sediments of aquatic ecosystems are at higher level compared with their concentration in corresponding water samples (Schweizer et al. 2018). The adsorption of pollutants on the sediments depends on both the particle size and composition of sediments especially the content of organic carbon (Milenković et al. 2005). The background level of Ln in river sediments varies significantly and depends mostly on the local geology. The source of metals in aqueous sediments could be both natural and anthropogenic, and it is important to distinguish the geochemical content of sediments from anthropogenic impact in order to further prevent environmental pollution. Sediments are regarded as a major sink of contaminants, but they can be also a source of contamination in desorption process (Roberts 2012).

The pollution of the second longest European river (the River Danube) has been under monitoring and focused on emerging contaminants including metals/metalloids (Hg, As, Ni, Zn, Cu, Cr, Pb, and Cd), personal care products, technical additives, pesticides, pharmaceuticals, steroids, and perfluorinated compounds (Milenković et al. 2005; Škrbić et al. 2018; Vuković et al. 2012; Ivanović et al. 2016; Comero et al. 2014; Reschke et al. 2002; Matić Bujagić et al. 2019). The biggest hydropower dam and reservoir system along the Danube River is Iron Gate I on Đerdap Gorge, 117 km long. The environmental impact of the dam includes alteration of the hydrological regime on the surface and ground waters and change of the sediment regime. The most important consequence of constructing the Iron Gate I is a constant increase of the amount of sediments (Babić Mladenović et al. 2013). The sedimentation rate within the Iron Gate I Reservoir is very fast (about 23.3 cm year^−1^) which implies a high potential of accumulation and as a consequence possibly preservation of pollutants (Micić et al. 2013). Because of the above facts, the Iron Gate I has been continuously in research focus due to the significant impact of the hydroelectric power station (Matić Bujagić et al. 2019, Micić et al. 2013; McGinnis et al. 2006; Teodoru and Wehrli 2005). There are several indications that the largest impoundments of the River Danube (Iron Gates) represent a kind of sink for pollutants (Reschke et al. 2002).

Although the occurrence, distribution, and fate of emerging contaminants including heavy metals in the Danube River have been well-examined and monitored, attention should be focused for those elements and compounds which are not monitored but start to appear in the environment and could be considered as future candidates for monitoring. Because of that, this study aims to determine major and trace elements including Ln in the Danube River sediment by instrumental neutron activation analysis (INAA). It should be emphasized that the INAA allows simultaneously determining more than 40 elements with high sensitivity due to specific nuclear reaction for each element and low detection limit. The INAA has the following advantages: a nondestructive method, the sample stays intact and no chemical separation treatment is involved, a small quantity of sample (≈ 100 μg), determination of the total element concentration independent of chemical species, real total analysis since the test portion does not have to be dissolved (Bode et al. 2017). The concentrations of the targeted elements in the surface sediments were discussed in the sense of the effect of building hydropower dam Iron Gate I and increasing the quantity of sediments in the Iron Gate Gorge.

## Materials and methods

### Description of the study area

The Danube River (2850 km long with the river basin of 801,463 km^2^) flows through 9 countries and geologically different areas. The Danube River in Serbia is 588 km long, from 1433 km (Hungarian border) to 845 km (border with Bulgaria), and flows through the three geomorphological areas (Vogel and Pall 2002; Woitke et al. 2003): reach 5 (from 1433 to 1202 km, the Pannonian part), reach 6 (from 1202 to 943 km, Iron Gate Gorge), and reach 7 (from 943 to 845 km, Lowland river). The sample places in this study are located in reaches 6 and 7. Reach 6 is characterized by the anthropogenic impact of the Iron Gate hydroelectric power plant and the significant inflow of untreated urban (especially form the city of Belgrade) and industrial wastewater, as well as agriculture effluents along upstream of the dam (Salvetti 2015). Reach 7 is characterized as the Lowland river (Aeolian sediments and loess). In the Serbian part, the River Danube receives two large tributaries (Rivers Sava and the Tisza) and several smaller ones (e.g. Velika Morava and Pek). Velika Morava is the last significant right bank tributary before the Iron Gates. River Pek is important because it passes close to the large dumpsites of a copper mine Majdanpek.

The main effect of hydroelectric plant construction (Iron Gate I) is the increase of sediment accumulation; more than 75% of the incoming sediment remain inside the reservoir (Teodoru and Wehrli 2005). Although the concentration of the suspended sediment is low (1–100 g m^−3^), as a consequence of big annual water flow (110–220 ×10^9^ m^3^), the quantity of sediments reaches 7–10 × 10^6^ tons (Babić Mladenović et al. 2013). The largest quantity of sediments (up to10 m high) was accumulated 3 years after the construction of the dam (1972–1974) and located between 970 and 1003 km of the River Danube. After that time, the sedimentation zone moved towards the dam. These sediments are built from suspended sediments of the Danube (39%), the Tisza (26%), the Sava (21%), and the Velika Morava (14%) (Babić Mladenović et al. 2013).

### Sampling and sampling locations

**Fig. 1:**
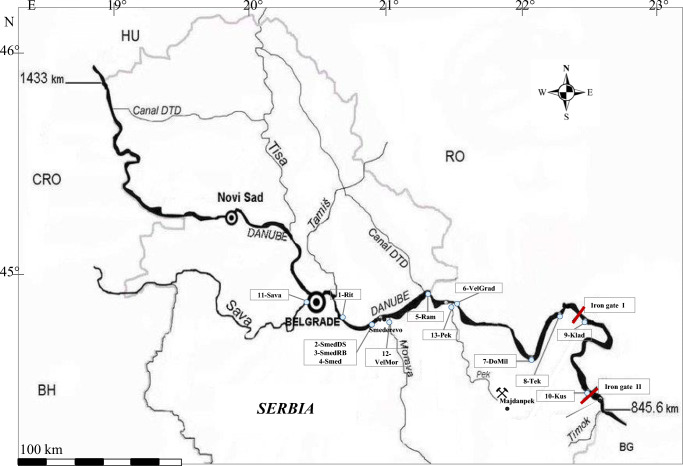
Map of the Danube River and its tributaries in the Republic of Serbia (sample locations are given in squares and explained in Table 1)

The samples were taken at 8 sites along the Danube River from 1141 to 864 km and in 3 tributaries (Fig. 1 and Table 1). Six samples belong to reach 6 and two samples to reach 7. We subdivided reach 6—the Upper stream from the Iron Gate I and the Iron Gate II—according to a different rate of sediment accumulation; significant accumulation of sediments in the Upper stream from the Iron Gate I is not pronounced. Sediments were collected from the surface of the river bottom at the central and the deepest part using an Ekman grab sampler in April 2016. The sediment samples are very fine with a strong muddy smell. Deep river sediment (1.5–2 m deep) and river bed (7.2–7.3 m deep) were collected at 1112 km of the River Danube. The samples (about 1 kg each) were transferred into the plastic containers and placed in a cooler for transport to the laboratory. The samples were mixed and air-dried in a thin layer in the dark at room temperature (23 ± 1 °C). After drying, the samples were homogenized using a mortar and pestle and sieved through a 1-mm sieve to ensure sample homogeneity.

**Table 1:** Properties of the sample locations

Sample code	River	River (km)	Place	Features (reach*)
1-Rit	Danube	1141	Ritopek	Upper stream from the Iron Gate I (reach 6)
2-SmedDS	1.5–2.0 m deep	1112	Smederevo	
3-SmedRB	7.2–7.3 m deep	1112	Smederevo	
4-Smed	Danube	1112	Smederevo	
5-Ram	Danube	1072	Ram	Iron Gate I (reach 6)
6-VelGrad	Danube	1059	V. Gradiste	
7-DoMil	Danube	991	D. Milanovac	
8-Tek	Danube	956	Tekija	
9-Klad	Danube	934	Kladovo	Iron Gate II (reach 7)
10-Kus	Danube	864	Kusjak	
11-Sava	Sava	5	Belgrade	Tributaries
12-VelMor	V. Morava	2	-	
13-Pek	Pek	0	-	

*Geomorphological reaches of the River Danube according to Vogel and Pall (2002)

### Quantification of the selected elements in the sediments

All samples are analyzed applying the INAA (Frontasyeva 2011; Greenberg et al. 2011). The major elements (Al, Ca, Fe, K, Na, Ti, Mg, and Mn), trace elements (Ba, Zn, Cr, Sr, V, Rb, Ni, Cu, Co, As, Sc, Th, Cs, Hf, Sb, U, W, Ta), and lanthanides (La, Ce, Nd, Sm, Eu, Gd, Tb, Dy, Tm, Yb) were quantified. Irradiations of the samples were performed at the pulsed reactor IBR 2 (Frank Laboratory of Neutron Physics—FLNP, Joint Institute of Nuclear research—JINR, Dubna, Russian Federation) using thermal or epithermal neutrons (Frontasyeva 2011).

Samples of sediments (≈ 100 mg each) were dried to constant mass at 100 °C and wrapped in polyethylene or aluminium foils for short- and long-term irradiations, respectively. For the determination of short-lived isotopes, samples were irradiated for 60 s and measured for 15 min by gamma ray spectroscopy using a high-resolution germanium detector (Canberra GC5519/7500SL). For quantification of long-lived isotopes, samples were irradiated for 4 days, repacked, and measured twice, after 4–5 days and after 20–23 days, with acquisition lasting for 30 min and 1.5 h, respectively. Data processing and determination of concentrations of the studied elements were performed using software developed in FLNP JINR (Pavlov et al. 2014).

In this study, we used a relative INAA and the concentration of analyzed element calculated according to the equation (Frontasyeva 2011):

1 $$ {C}_{\mathrm{sample}}={C}_{\mathrm{stand}}\frac{A_{\mathrm{sample}}}{A_{\mathrm{stand}}} $$

where *A*_sample_ and *A*_stand_ are activities measured in samples and standards (Certified Reference Materials—CRM), and *C*_sample_ and *C*_stand_ are corresponding concentrations. CRM (IRMM BCR 667-estuarine sediment), NIST SRMs 2709 (trace elements in soil), and 1632c (trace elements in coal bituminous)) for irradiation is prepared using the same procedure as the samples. Also, the accuracy of the applied method was checked and confirmed by the analysis of CRM. All measurements were done in triplicate, and the mean concentration was used for further analyses. Relative standard deviations of measurements range from 3.8 to 14.7%, except for Gd and Dy (RSD = 25%). The chemical matrix effect is known to be significant sources of error in many instrumental chemical analyses, but it is insignificant in the INAA (Greenberg et al. 2011).

### Determination of total organic carbon

Total organic carbon (TOC) was determined by a semi-quantitative method which involves thermal destruction of all organic matter in the samples. Sample of known weights (previously heated at 100 °C) was placed in a ceramic vessel and heated at 400 °C until the constant weight (2 h). Then, samples were cooled in a desiccator and weighted at analytical balance. TOC is calculated as the difference between the initial and final sample weight (Bisutti et al. 2004).

### Multivariate analysis

Multivariate analysis and chemometric methods were applied to better understand the spatial distribution of the quantified elements and to highlight hidden relationship between variables, i.e. concentrations and sample locations. Principal component analysis (PCA), factor analysis, and hierarchical cluster analysis (HCA) were carried out by Minitab 16.1.0. software (2010, Minitab Inc., State College, PA). All data were autoscaled prior to multivariate analysis. The more details are given in the supplementary material.

## Results and discussion

Thirty-six elements were determined in the studied sediments of the River Danube and three tributaries applying the INAA. All measurement data including the descriptive statistic results (mean concentration and standard deviation) are given in Table S1 in the supplementary material. The concentrations of studied elements in the monitoring sediments are very variable between the sample locations with relative standard deviation from 12.5 to 140%. The highest variability (RSD > 30) was found for Cu, Na, Ca, Zn, Cr, Ni, As, Cs, and Sb.

### Major, minor, and trace element composition in the surface and deep sediments

The most abound metals (major elements) in the sediments, ranging from 0.12 to 9.9%, that we found are Al, Fe, K, Na, Ca, Ti, Mg, and Mn (Table S1a in the supplementary material). The highest variability in the group of major elements (RSD > 30) was observed for Ca and Na, e.g. Ca concentration is significantly higher in the samples 11-Sava and 1-Rit which are close each other with similar landscape. The highest concentration of Na and the lowest concentration of Ca were found in two tributaries (12-VelMor and 13-Pek) because of their different geochemical properties. The highest contents of Al, Ti, Mg, and Mn, as well as TOC, were found in the samples located in the Iron Gate I as a consequence of a decrease in water flow rate which facilitates particle sedimentation (Teodoru and Wehrli 2005). The concentrations of the major elements in the samples within the Iron Gate I agree with those obtained in the study of Kašanin-Grubin et al. (2020) at 1030 km. Lower concentrations for almost all major elements were found in sample 1-Rit which is far away from the Iron Gate I and at the border with reach 5 which has different geochemical content compare to other investigated Danube sediments. Only Fe and K are homogeneously distributed in the surface sediments of the Danube, while their concentrations are lower in tributaries and deep sediments. Analysis of the deep sediments (2-SmedDS and 3-SmedRB) shows lower concentration levels for almost all determined elements. Only Ca and TOC are particularly pronounced in the sample at a depth of 7 m (3-SmedRB) probably due to long-time precipitation of the residues of plants and animals decay.

**Fig. 2:**
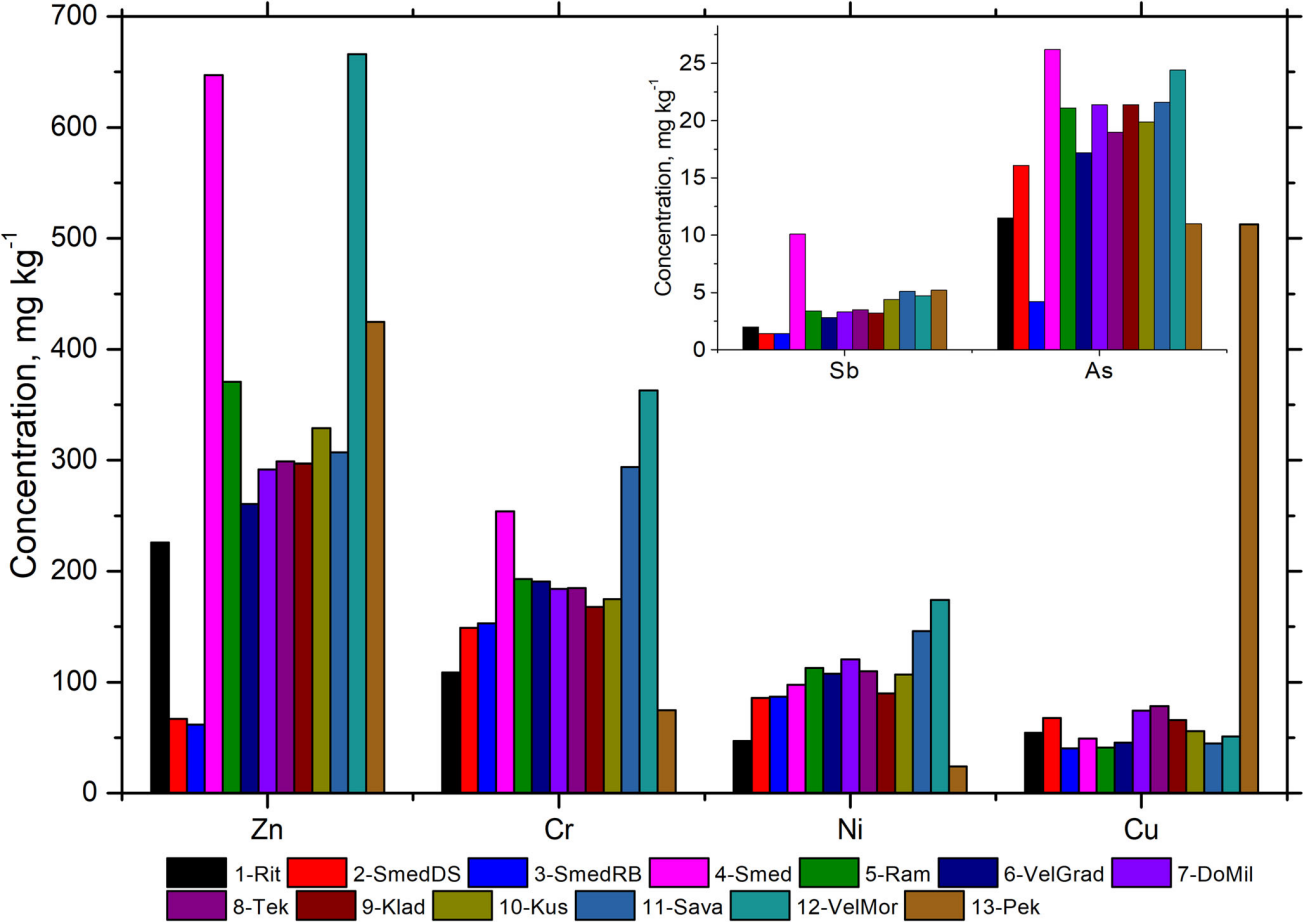
Concentrations of the minor and trace elements measured in the surface and deep sediments of the river Danube and its tributaries

Concentrations of all other measured elements are below 0.1% (minor and trace elements, Table S1b in the supplementary material). Spatial distribution of the elements with variability within groups higher than 30% is presented in Fig. 2. In order to deeply evaluate contamination of the sediments, the determined concentration of the elements can be compared with background concentration either from sediment cores or pristine sediments or average shell concentration (Woitke et al. 2003; Abrahim and Parker 2006). We used the values from the sample 3-SmedRB as background concentration because this sample was taken from 7 m depth and the lowest concentrations of almost all elements were found in it. Enrichment factor (EF) was calculated applying the following equation:

2 $$ \mathrm{EF}=\frac{\left[{X}_i\right]:\left[{X}_{3-\mathrm{SmedRB}}\right]}{\left[{Fe}_i\right]:\left[{\mathrm{Fe}}_{3-\mathrm{SmedRB}}\right]} $$

where [*X*_*i*_] and [Fe_*i*_] are concentrations of element *X* and Fe at sampling location *i*, [*X*_3-SmedRB_] and [Fe_3-SmedRB_] are concentration of element *i* and Fe in the sample 3-SmedRB. Iron was chosen as a normalizing element because the concentration of Fe is the most homogenous in this study, and Fe concentration in 3-SmedRB agrees well with background concentration in Fossil Rhine Sediments (Förstner and Müller 1981), Upper Parramatta River Catchment Australia (Birch et al. 2000), and Canadian background levels (Grosbois et al. 2001). Assuming that EF > 2 indicates anthropogenic pollution (Woitke et al. 2003), Table 2 shows the studied elements at specific sampling places with EF > 2.

**Table 2:** Maximum enrichment factors (EF) for minor and trace elements in the studied sediments of the River Danube and its tributaries normalized with respect to the iron content in sample 3-SmedRB

Element	EF											
	1-Rit	2-SmedDS	4-Smed	5-Ram	6-VelGrad	7-DoMil	8-Tek	9-Klad	10-Kus	11-Sava	12-VelMor	13-Pek
Zn	3.7	1.1	8.1	4.3	3.3	3.3	3.4	3.4	3.7	4.0	8.1	7.4
Cu	1.4	1.7	0.9	0.7	0.9	1.3	1.4	1.2	1.0	0.9	1.0	13.8
Sb	2.8	3.9	4.8	3.6	3.3	3.5	3.1	3.7	3.3	4.2	4.4	2.8
As	1.4	1.0	5.6	1.7	1.6	1.6	1.7	1.6	2.2	3.0	2.5	4.0

The highest EF (13.8) and concentration, higher than remediation value prescribed by national law (Table 3) (Sl. Glasnik RS 2012), were found for Cu in the sediments of the River Pek (13-Pek). This river flows near the Majdanpek copper mine and that is the reason for the high concentration of Cu. EFs of zinc and antimony ranging from 2.8 to 8.1 were estimated for all investigated surface sediments which means that these EFs are moderate and moderately severe and indicate their anthropogenic origin (Elias et al. 2018). The concentration of Zn in the samples 4-Smed and 12-VelMor exceed the maximum allowed values; therefore, all other samples are higher than target values but below the maximum allowed values (Table 3). Although the EF of arsenic in some samples reaches a value of 5.6, the concentration of As is below the target values in all samples (Table 3). The sediment sample of the River Danube, 4-Smed, is a hotspot of contaminations with Zn, Cr, and As, which indicates an anthropological influence probably derived from a steel processing plant which is located near the town of Smederevo. It should be noted the concentration of Ni is above maximally allowed concentration in all samples except for 13-Pek (Table S2 in the supplementary material), but corresponding EFs are lower than 1.5. Also, concentration of Cr is higher than maximal allowed concentration in the surface sediment of 4-Smed, 11-Sava, and 12-VelMor. This shows that the impact of different elements on pollution should be analyzed comprehensively. Other investigated trace elements (Sc, V, Rb, Sr, Cs, Ba, Hf, W, and Th) are very uniform in the sediment belonging to reaches 6 and 7, whereas the concentration is lower in deep sediments and tributaries. Ta and U represent elements with the lowest variability among all investigated samples.

**Table 3:** Limit values (in mg kg^−1^) for assessing the status and trend of sediment quality according to the regulation on limit values of pollutants in surface and groundwater and sediment (“Sl. glasnik RS”, br. 50/2012)

Element	Target value	Maximum allowed	Remediation value
As	29	42	55
Cd	0.8	6.4	12
Cr	100	240	380
Cu	36	110	190
Hg	0.3	1.6	10
Pb	85	310	530
Ni	35	44	210
Zn	140	430	720

Comparison between the heavy metal concentrations in different time periods from 1985 to 2016 (Table 4) shows a trend of increasing concentrations of Fe, Mn, Zn, Cr, Ni, and Cu in the investigated surface sediments (Milenković et al. 2005). The increase of Cr concentration in sample 4-Smed, which is 5 times higher in 2016 than in 2002, is especially pronounced and indicates an anthropological influence—probably derived from a steel processing plant as we mentioned above.

**Table 4:** Concentrations of the selected elements in the sediments of the River Danube quantified in 1985*, 2002*, and 2016**

Sample location	Zn (mg kg^−1^)			Cr (mg kg^−1^)			Ni (mg kg^−1^)			Cu (mg kg^−1^)		
	1985	2002	2016	1985	2002	2016	1985	2002	2016	1985	2002	2016
4-Smed	-	219.1	647	-	51.8	254	-	46.8	98	-	23.9	49.4
5-Ram	-	328.4	371	-	112.5	193	-	116.4	113	-	36.8	41.1
6-VelGrad	230	389.5	261	54	105.9	191	39.0	99.9	108	25	41.0	45.5
7-DoMil	-	285.7	292	-	68.0	184	-	69.9	121	-	45.3	74.6
8-Tek	210	307.8	299	50	93.3	185	34.0	74.5	110	35	57.6	78.7
9-Klad	-	197.5	297	-	71.1	168	-	59.2	90	-	31.6	66.2

*(Milenković et al. 2005)

**This study

### Composition of the studied Ln in the surface and deep sediments

Measured concentrations of Ln in the investigated sediments are presented in Table S1c and Fig. S1 in the supplementary material. The concentration range of Ln ranges from 0.3 to 71 mg kg^−1^. Based on the descriptive statistic of the investigated Ln (Table S1c), estimated RSD range was from 10.5 to 29.6%. Yb and Tb are the most uniformly distributed elements in the studied samples with RSD 10.5% and 12%, respectively. Contrary, Nd, Sm, and Dy are the elements with the highest variability in the studied sediments with RSD > 20%. The mean value of total Ln concentration is 146.13 ± 20.60 mg kg^−1^and little higher than the corresponding value (124.36 mg kg^−1^) obtained for river clay fraction of the Danube Delta (Bayon et al. 2015). Lower ΣLn was found in two sediments: 4-SmedRB and 11-Pek.

**Fig. 3:**
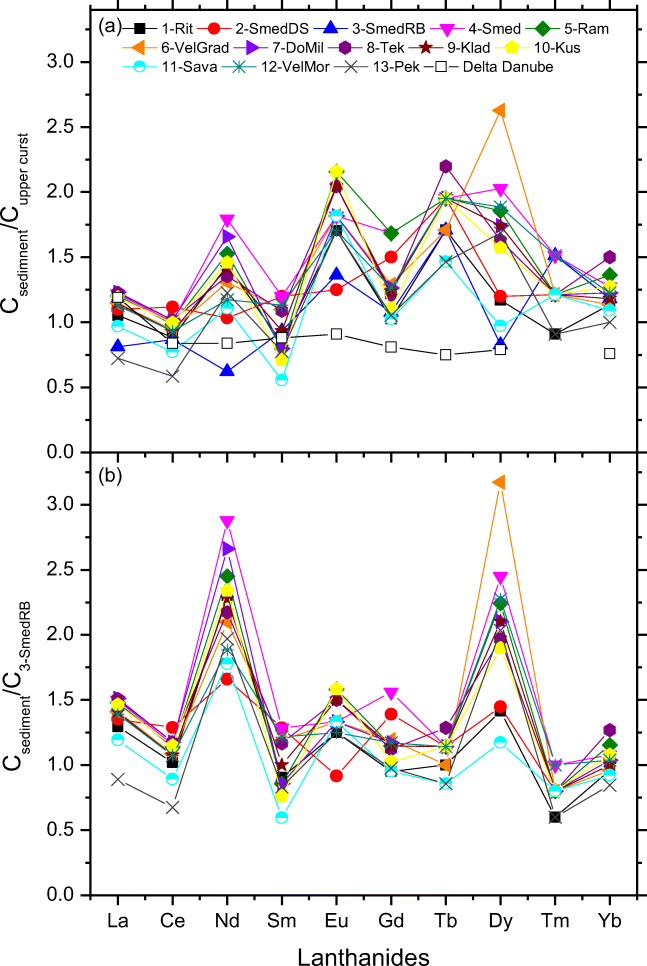
Normalized Ln distribution pattern in the sediments of the River Danube and its tributaries against (**a**) Upper crust concentrations (Taylor and McLennan 1985), and (**b**) concentration in sample 3-SmedRB. Black open square represents the normalized values of Ln in clay fraction of the Delta Danube sediment in Romania (Bayon et al. 2015)

Given the large difference in Ln concentrations in nature compartment, a pattern of the rare earth elements is usually described by normalizing these element concentrations in the sample to those of the crustal abundance of the earth. We applied two approaches for lanthanide normalization: (1) against relative concentration in Upper crust concentrations taken from Taylor and McLennan (1985) (Fig. 3a); (2) against concentration in sample 3-SmedRB. The second approach is based on lower quantified concentration of all studied Ln in sample 3-SmedRB in comparison to surface sediments of the River Danube as well as this sample was dug on 7.2–7.3 m deep; thus, it can be assumed as unpolluted, and used to highlight the possibility of anthropological influence (Fig. 3b).

The spatial distribution of Ln normalized against upper crust concentration ranges from 0.6 to 2.6 (Fig. 3a). Normalized values > 1.5 were found for Eu, Tb, and Dy in almost all studied surface sediments. The obtained patterns have pronounced maxima, while, for example, the normalized REE distribution pattern in the river sediments in Korea and China has differences between heavy and light Ln (Xu et al. 2009). We show in Fig. 3a the normalized values of Ln in clay fraction of the Delta Danube sediment in Romania (Bayon et al. 2015). The lower mean normalized values (0.86 ± 0.13) were obtained probably because of different geological properties and only clay fraction was analyzed. Mean normalized values of all Ln in the sample 3-SmedRB is 1.12 ± 0.35. This is an additional reason to choose this sample as pristine and in further work applied for normalization of other samples.

Figure 3b depicts the spatial distribution of relative concentration of Ln in the surface sediments calculated against their concentration into deep river sediments (3-SmedRB). By comparing Fig. 3a and b, difference of the normalized pattern of Ln in the case of differently applied reference values for normalization is clearly visible. In the second approach (Fig. 3b), high homogeneity for almost all samples was obtained. The largest differences were observed for Nd and Dy, even 3 times higher for the sample 6-VelGrad (Fig. 3b). It is obvious that the second approach, which resulted in much better outcome in terms of uniformity and clarity due to similar matrix composition, confirms that potentially unpolluted sample of river bed (sampled at 7 m) is a better choice for matrix match (Woitke et al. 2003). Since this is the first analysis of Ln in the sediments of the River Danube, the sources of potential pollution are difficult to identify. We can assume that the sources can be effluents from untreated urban and industrial waste waters near the sampling sites. Also, air pollution caused by burning coil lignite, which represents the largest air pollution in Serbia, can be the source of Ln (Franus et al. 2015).

### Chemometric evaluation of total pool of measurements for all elements

For better understanding and to highlight a hidden relationship between targeted elements, multivariate statistical techniques were applied to analyze the analytical data and to identify possible pollution sources. It is well-known that environmental data are strongly characterized by inherent variability and many variables must be considered. PCA and HCA have been applied to assess the level of heavy metals in sediments (Wang et al. 2019; Huang et al. 2020), soil (Siddiqui et al. 2020), etc. PCA is used to reduce dataset to a small number of independent components for analyzing relationships among the observed variables. HCA has been applied to identify different geochemical groups, clustering the samples on the basis of the similarities of their chemical properties (Yongming et al. 2006; Ražić 2011).

**Fig. 4:**
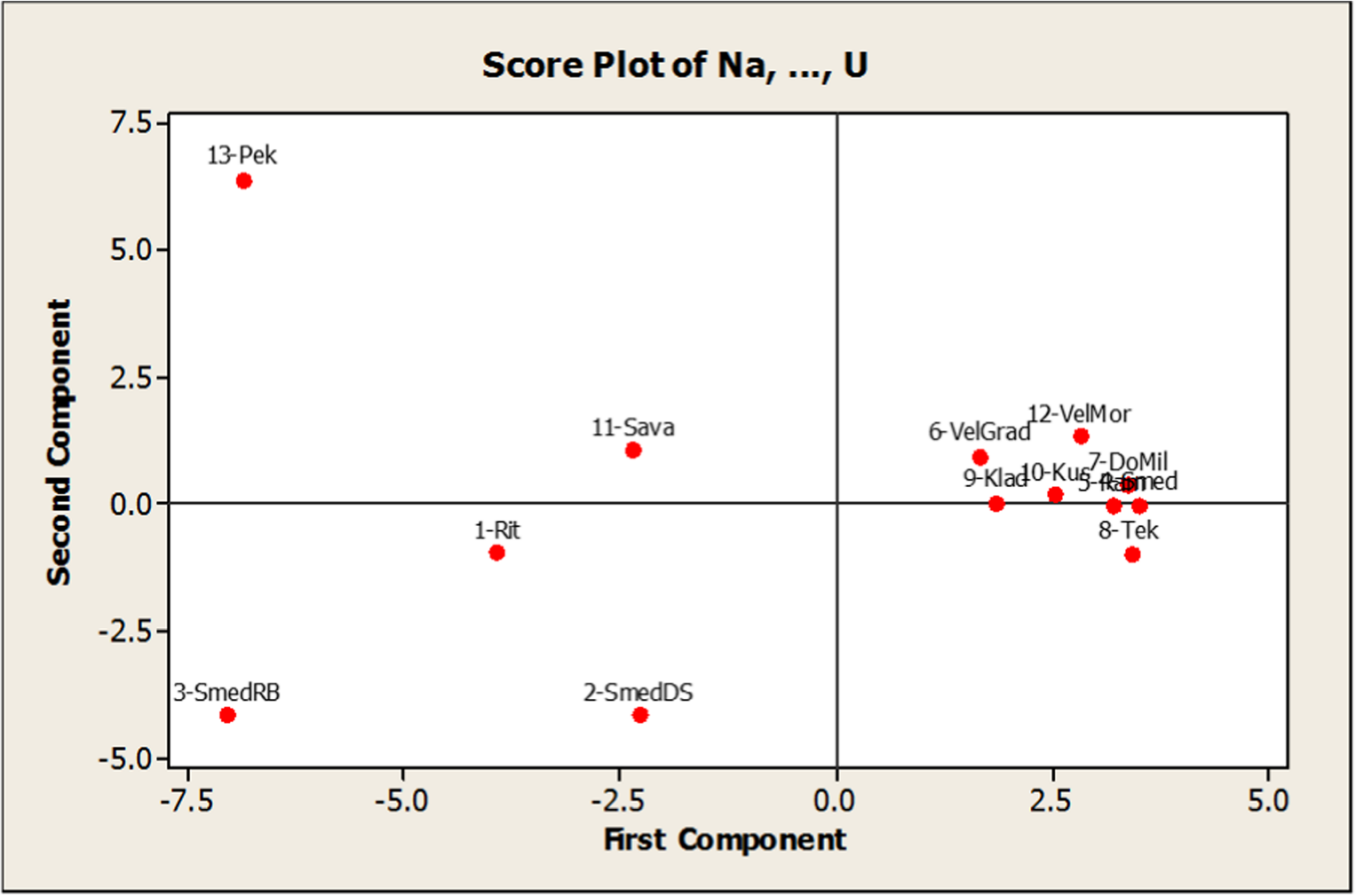
Score plot of PCA in analysis of the measured elements in regard to the sample locations

The data set of concentration measurements was subjected to a PCA in order to decrease the number of descriptors responsible for the highest percentage of the total variance of the experimental data. When PCA was applied to the autoscaled data matrix, with Eigen analysis as an initial (Table S2 and Fig. S2 in the supplementary material), six principal components (PCs) were extracted according to the Kaiser criterion which explains up to 93.80% of variance. Thus, the true dimensionality of the descriptor space is six. As a result, we have got a space that can be described with six factors. Features with high positive or negative loadings essentially determine the factor. Since there were too many medium factor loadings in the first (unrotated) matrix for a solution of the factor, the mathematical rotation appeared as necessary to simplify the structure of the factors for a better interpretation. We applied the Varimax rotation (Table S3 in the supplementary material). In this way, by extracting the six PCs, we managed to reduce the contribution of variables with minor significance. Factor scores are derived from the PCs and are specific to individual objects, i.e. locations in our case, not to variables (visualization of the obtained results is presented in Fig. 4 and Fig. S3 in the supplementary material). There are differentiated 13-Pek (higher Cu) and 1-Rit, 2-SmedDS, and 3-SmedRB (higher Ca). The elevated Cu is derived from close vicinity of the copper mine. The second group, to the right, in the direction of increased all other investigated elements including Ln, lies in the area of the Iron Gate Gorge. The elevated concentrations of these elements can be explained by the increasing deposition of sediments.

**Fig. 5:**
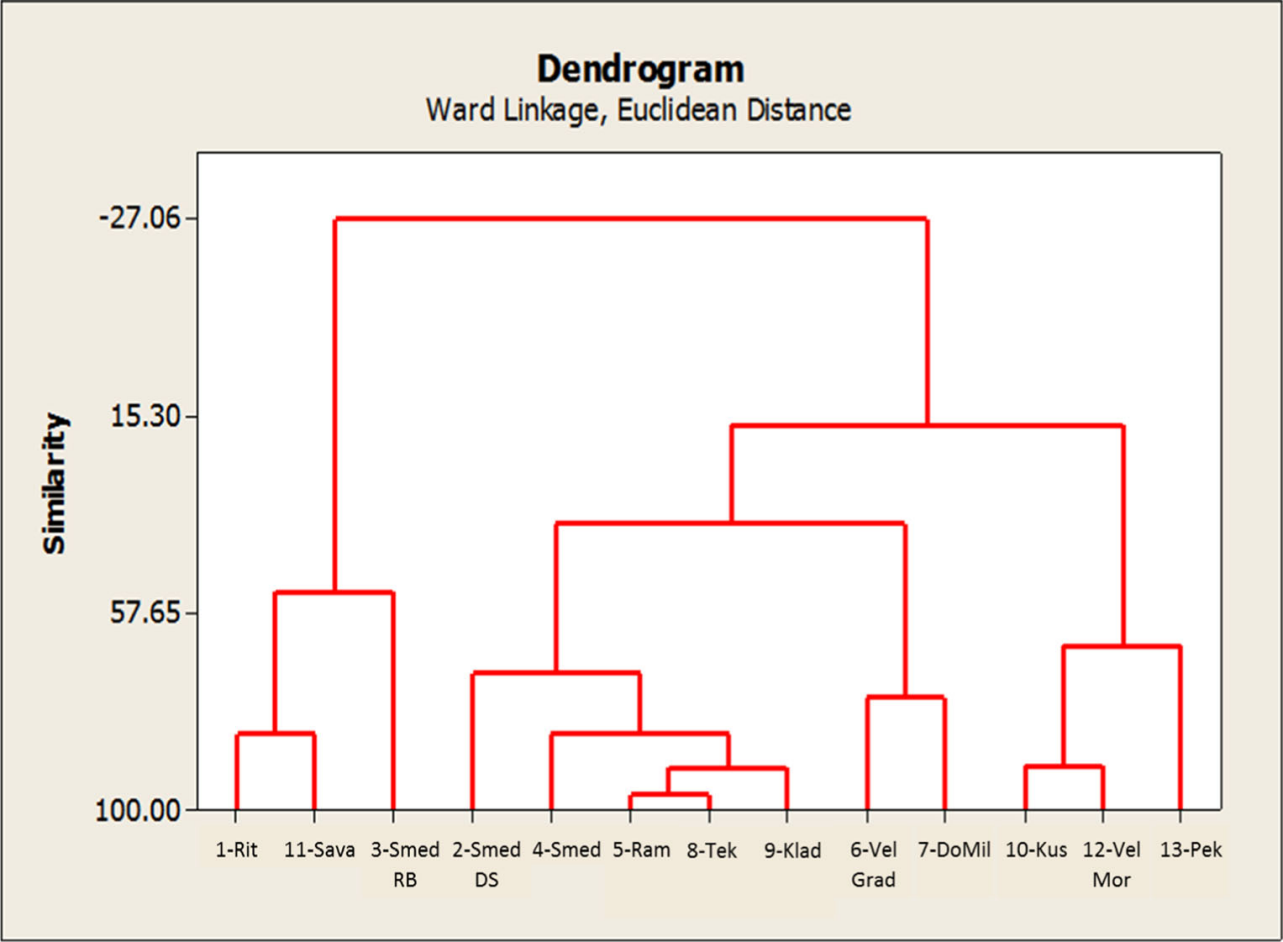
Dendogram presenting hierarchical clustering of sampling locations. Analysis includes all determined elements

Going further with chemometric evaluation to identify relatively similarity, i.e. homogenous groups of objects (locations) in the space of measured features (elements), a hierarchical agglomerative cluster analysis (HCA) was performed. Ward’s method, as an amalgamation rule, was applied on standardized concentrations of the elements and the Euclidian distance as a measure of the proximity between samples. Well-differentiated corresponding dendrogram is shown in Fig. 5. The first cluster comprises sub-cluster with samples 1-Rit and 11-Sava, geographically close to each other and sample from location 3-SmedRB, with similar concentrations for majority of elements (Table S1 in the supplementary material). It should be emphasized that 3-SmedRB is distanced from all other samples (Fig. 4) because its only sample which excavated at a depth of 7.2 m. The second cluster comprises the samples belonging to surface sediments of the River Danube from 1112 to 934 km which is characterized with increased accumulation due to the building of the dam. Sample 2-SmedDS is a bit separated (Fig. 4) and also at the edge of the 2nd cluster (Fig. 5) because the sampling was done from a deeper level (2 m). Samples from locations 10-Kus and 12-VelMor belong to the third cluster with an assumption of similar concentrations for Ca, V, and Sb (Table S1 in the supplementary material). Location 13-Pek is already discussed concerning the extremely high concentration of Cu due to the vicinity of the copper mining site. In certain cases of chemometric evaluation, such extreme values are being discarded as outliers to enable further modelling, but in this work, we retained it based on knowledge of its source.

### Chemometric evaluation of the studied Ln

In order to get a better insight into the Ln profile in analyzed samples, the dataset was subjected to the second round of PCA. Following the methodology explained in the first part—the analysis of all measured elements—the data set of Ln concentrations was subjected to a PCA, with Eigen analysis as an initial, when three PCs were extracted explaining 84.95% of variance. In this case, the true dimensionality of the descriptor space is three (Table S4 a–c).

**Fig. 6:**
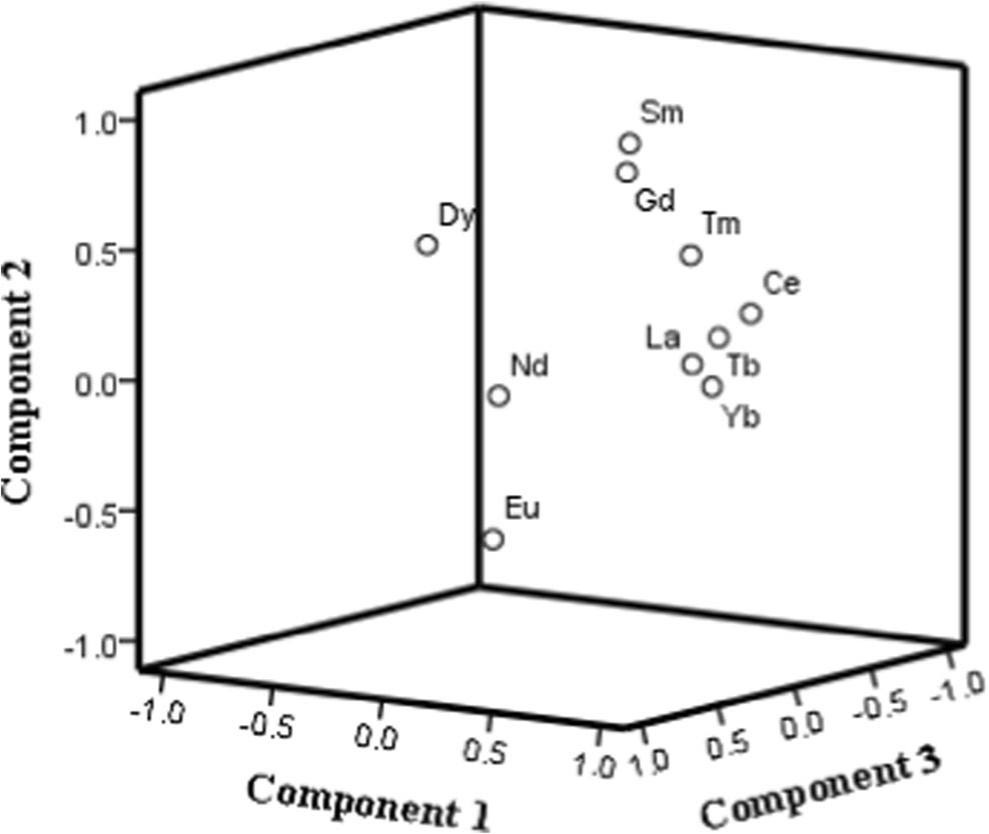
Component plot in rotated space

The score plot of the studied Ln derived from the PCA is presented in Fig. S4 in the supplementary material. As aforementioned, the sample 3-SmedRB from a deep river bed (7.2 m deep) is separated from other samples on a score plot of Ln, and because of that, it was assumed as intact and unpolluted (without anthropological influence). From that reason, in further study, the normalized concentration of Ln against the concentrations of Ln in the sample 3-SmedRB were used to the third round of PCA (Fig. 6). In line with discussion on impact of variability of concentrations in dependence of location, its strong influence was confirmed through PCA. The separation of Dy and Nd in regard to other Ln is obvious (Fig. 3b and Fig. 6).

**Fig. 7:**
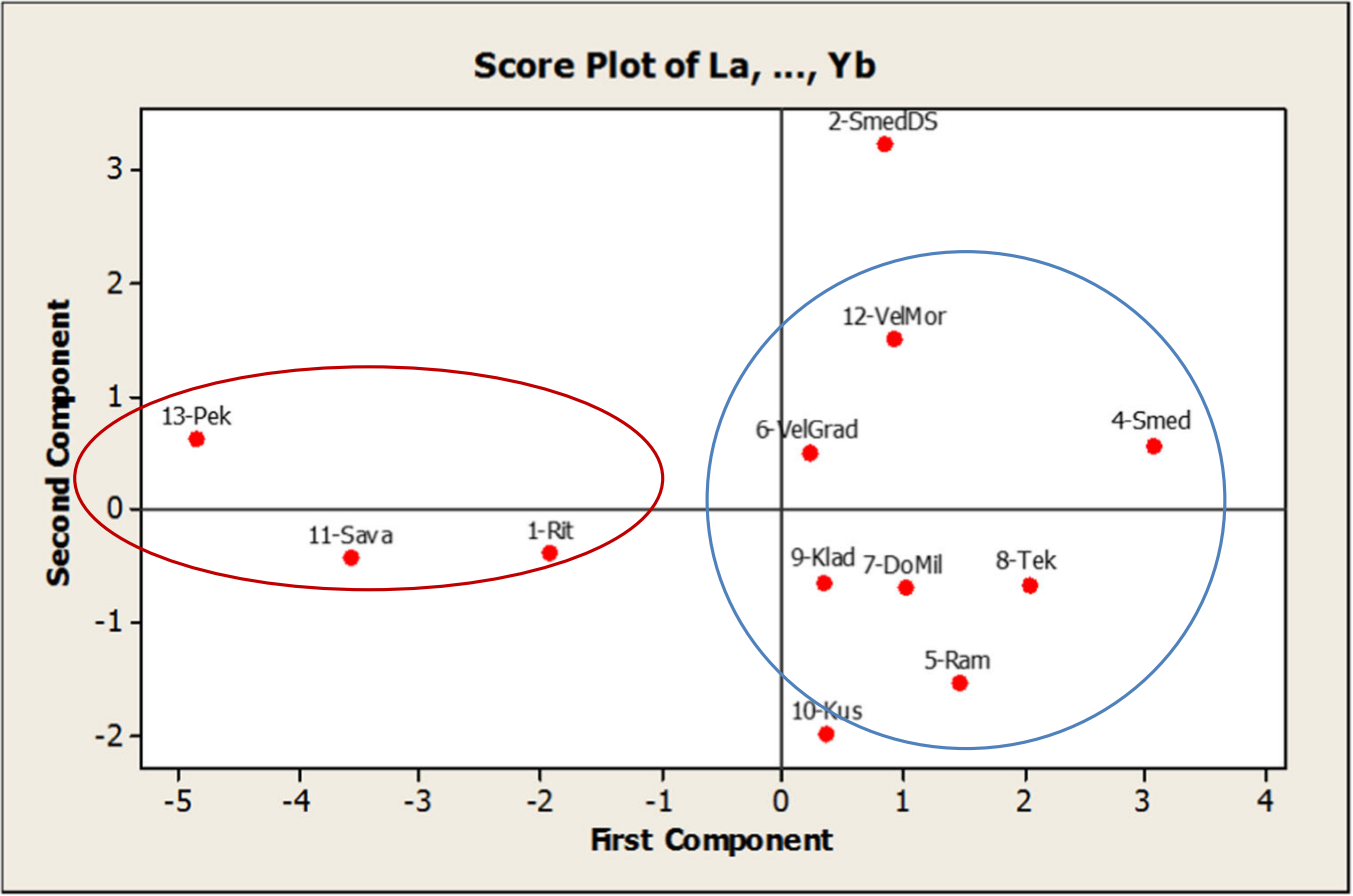
Score plot of PCA of normalized concentrations of Ln in regard to the sampling locations

All the obtained results on Ln concentration with its inherent variability produced the possibility of visualization by score plot, taking into account the locations which were measured (Fig. 7). Grouping of samples1-Rit, 11-Sava, and 13-Pek (red circle) is obviously the result of lower total concentration of all Ln, presented as ΣLn in Table S1c in the supplementary material. Another group (blue circle) is represented by those samples with increased concentration of Ln. The sample 2-SmedDS, which was excavated at a depth of 1.5–2 m, exhibits the lowest variability of Ln in comparison with other samples (Fig. 3), and from that reason, it is a bit separated from other locations in Fig. 7.

## Conclusion

This study provides new information about the distribution of 36 elements, including Ln for the first time, in surface sediments of the River Danube collected from 1141 to 864 km and three major tributaries on this stream. The studied elements exhibited high spatial variabilities, with the highest concentrations in the samples located in the Iron Gate I region, where the accumulation of sediments is pronounced as a consequence of building the dam. The extremely high concentration of copper, higher than remediation value prescribed by national law, was found in the sediments of River Pek that flows near the Majdanpek copper mine. The sediment sample of the River Danube, 4-Smed, is a hotspot of contaminations with Zn, Cr, and As, which indicates an anthropological influence probably derived from a steel processing plant which is located near the town of Smederevo. We found that the levels of Zn, Cr, and Ni are significantly higher compared to earlier data due to increasing accumulation of sediments as the consequence of building the Iron Gate I dam.

The concentrations of the studied Ln in the Danube surface and deep sediments were determined for the first time, and the obtained data are important in the creation of a database necessary for tracking anthropogenic intrusion on the content of natural objects. The normalized pattern of Ln in the investigated sediments show maximum which implies anthropogenic influence. Also, this pattern depends on applied approach for normalization as intact and unpolluted. We found use of the deep river bed sample (3-SmedRB) for normalization gives more homogeneous Ln patterns. The impact of variability of concentrations in dependence of location was confirmed by PCA and visualization of the separation of Dy and Nd in all sediments belonging to the Iron Gate Gorge.

Since this is the first analysis of Ln in the sediments of the River Danube, the sources of potential pollution are difficult to identify. We can assume that the sources can be effluents from untreated urban and industrial waters near the sampling sites. Also, air pollution caused by burning lignite, which is the largest pollution in Serbia, can be the source of Ln. Considering the wide application of Ln as technological critical elements and ecological importance which is still not well-estimated, further work should focus on periodic monitoring of these elements in surface sediments as well as corresponding river water to deeply understand their sources and fate in environment. It is necessary to emphasize the importance of a multivariate approach in the study of Ln. With the obtained experimental results and applied chemometrics tools, we demonstrated the possibility of clear distinction of locations according to the Ln profile.

## Supplementary information

ESM 1 (DOCX 199 kb)

## Data Availability

The datasets obtained and analyzed in the current study are available from the corresponding author on reasonable request.
