# Supplementary material for: Spatial distribution of multielements including lanthanides in sediments of Iron Gate I Reservoir in the Danube River
Source: Environ Sci Pollut Res Int. 2021 Apr 14;28(33):44877–89. doi: 10.1007/s11356-021-13752-6 (PMC8364546; doi:10.1007/s11356-021-13752-6)
Supplement: Supplementary file 1 — (DOCX 199 kb) [file 11356_2021_13752_MOESM1_ESM.docx]

**Supporting information**

**Spatial Distribution of multielements including lanthanides in sediments of Irong Gate I Reservoir on the Danube River**

Otilia Ana Culicov^1,2^, TatjanaTrtić-Petrović^3*^, Roman Balvanović^3^, Anđelka Petković^4^, Slavica Ražić^5^

*^1^Frank Laboratory of Neutron Physics, Joint Institute for Nuclear Research, Dubna, Russian Federation*

*^2^National Institute for R&D in Electrical Engineering ICPE-CA, Bucharest, Romania*

*^3^Laboratory of Physics, Vinča Institute of Nuclear Sciences, National Institute of the Republic of Serbia, University of Belgrade, Belgrade, Serbia*

*^4^“Jaroslav Černi” Institute for the Development of Water Resources, Belgrade, Serbia*

*^5^University of Belgrade, Faculty of Pharmacy - Department of Analytical Chemistry, Belgrade, Serbia*

^*^Corresponding author address:

*Laboratory of Physics, Vinča Institute of Nuclear Sciences, National Institute of the Republic of Serbia, University of Belgrade, P.O. Box 522, 11001 Belgrade, Serbia,* [*ttrtic@vin.bg.ac.rs*](mailto:ttrtic@vin.bg.ac.rs)

**Details about performed multivariate analysis**

The reduction of high dimensional feature space, to one which can be explained with fewer variables, is important to highlight the significant correlations, usually hidden in the original dataset. A data matrix, with locations presented in rows and elements as descriptors in columns, was built for further multivariate analysis (Ražić 2011). The Ryan-Joiner test was applied to examine whether or not the experimental data follow a normal distribution. Variables (features) were standardized (transformed) for further processing because of different units and ranges of measurements. The mean value and standard deviation are frequently used for this purpose and each value (x_i_) is replaced by the corresponding z_i_ value using the expression:

 (2)

where z_i_ is the autoscaled value of x_i_ and SD is the standard deviation of variable x.

Then, a data matrix, with locations presented in rows and autoscaled values of elements’ concentration as descriptors in columns, was built for further multivariate analysis. The obtained data set was subjected to correlation analysis. Exploratory approach of Factor analysis was applied.

***Factor analysis***

Factor analysis is one of the often-used methods for analyzing multivariate data. It explains observed multivariate data in terms of the linear relationships between a smaller number of unobserved variables, called latent variables or factors. Factor Analysis is based on an assumption that these factors exist and that their number is exactly known, so the method is reliable only if the modeled system is well understood. Contrary to this, principal component analysis does not rely on such assumptions about covariance matrix and uses linear combinations of the observed variables. We employed both methods to cross check the produced results (Ražić 2011).

An initial statistics of the correlation matrix was done using Eigen analysis. Eigen values are calculated according to the expression:

$R\cdot e_{1}=\lambda\cdot e_{1}$ (3)

where R is the correlation matrix, e are eigen vectors and λ are eigen values.

The eigen values are measure of the extracted variance from the total feature variance s2 total. The sum of the eigenvalues is equal to the number of features (variables). The total number of features describes the variance of the full data set (numerical patterns – loadings). We gave an advantage to Kaiser criterion over Catell’s and retained only the first components with subsequent eigenvalues less than one since they accounted for a high percentage of the determinable variance.

After extraction of the factors, the obtained matrix will show the significant loadings in each factor and present the combination of variables. That means that principal component analysis (PCA) transformed a set of possibly correlated random variables into a set of uncorrelated variables, called principal components. Using PCA, the reduction of the data set is realized by transforming the data into orthogonal components that are linear combinations of the original variables. This way, by compression, PCA reduces the complexity of high-dimensional data while retaining its patterns. Furthermore, when the obtained matrix contained too many medium factor loadings it appeared to be echnique to interprete the solution of the factors. That’s why we applied a rotation of the coordinate system of factors since it does not affect the position of the objects (locations) relative to each other, but will simplify the structure of the factors. Among several available numeric transformation algorithms we selected varimax because it minimized the number of variables with high absolute values of factor loadings.

***Cluster analysis***

Another unsupervised learning method that belongs to Factorial methods is cluster analysis (CA) (Ražić 2011). This method is very powerful in visualizing structural similarities (groups, classes) in the data and can be seen as a pattern cognition method.

For finding structures in a data set, there is a necessity for similarity (or distance). This is derived from geometry. The Euclidean distance of any two objects A and B is calculated according to expression:

$d(i,k)=\sqrt{\sum_{i=1}^{m} (x_{ij}-x_{kj})^{2}}$ (4)

where m is the number of features (variables) and d(i,k) is the Euclidean distance of any two objects A and B. In the obtained distance matrix the correlation coefficients appear as a measure of similarities of each pair of features, instead of distances between objects. In selecting strategy, hierarchical echnique was applied because the typical output of a hierarchical clustering method is dendrogram, as very helpful in interpreatation of our results. As the mode of the agglomerative hierarchical method the Ward linkage was selected.

Reference:

Ražić S (2011) Chemometrics in the Analysis of Real Samples - From Theory to Application. Ed. Faculty of Pharmacy - University of Belgrade, ISBN 978-86-80263-81-6

**Table S1a**. Experimental (in mg kg^-1^ and in %) and statistic data (mean concentration, standard deviation (SD) and relative standard deviation (RSD)) of the quantified major elements and total organic carbon (TOC) in the sediments at 13 monitoring sites along the River Danube and three tributaries.

| Sample  code | Concentration of major elements, mg kg^-1^(%) | | | | | | | | TOC, % |
| --- | --- | --- | --- | --- | --- | --- | --- | --- | --- |
|  | Al | Ca | Fe | K | Na | Ti | Mg | Mn |  |
| 1-Rit | 56900 (5.7) | 70800 (7.1) | 33100 (3.31) | 16200 (1.6) | 9090 (0.9) | 5050 (0.51) | 3110 (0.31) | 1250 (0.13) | 4.41 |
| 2-SmedDS | 64700 (6.5) | 52300 (5.2) | 33300 (3.33) | 14100 (1.4) | 5940 (0.60) | 4320 (0.43) | 5330 (0.53) | 704 (0.07) | 3.20 |
| 3-SmedRB | 48800 (4.9) | 99500 (9.9) | 33500 (3.35) | 13400 (1.3) | 5880 (0.59) | 3310 (0.33) | 4190 (0.42) | 1460 (0.15) | 8.29 |
| 4-Smed | 68400 (6.8) | 60600 (6.1) | 43400 (4.34) | 20300 (2.0) | 7870 (0.8) | 5300 (0.53) | 3210 (0.32) | 1220 (0.12) | 6.15 |
| 5-Ram | 75500 (7.6) | 53200 (5.3) | 47100 (4.71) | 21100 (2.1) | 7370 (0.7) | 5380 (0.54) | 3470 (0.35) | 1650 (0.17) | 7.77 |
| 6-VelGrad | 99400 (9.9) | 68700 (6.9) | 42200 (4.22) | 20100 (2.0) | 9310 (0.9) | 8270 (0.83) | 4680 (0.47) | 1890 (0.19) | 7.54 |
| 7-DoMil | 90100 (9.8) | 53000 (5.3) | 48400 (4.84) | 20500 (2.1) | 6920 (0.7) | 6660 (0.67) | 3740 (0.37) | 1930 (0.20) | 7.03 |
| 8-Tek | 75200 (7.5) | 55300 (5.5) | 48200 (4.82) | 21400 (2.1) | 7340 (0.7) | 5720 (0.57) | 3740 (0.37) | 1540 (0.15) | 7.94 |
| 9-Klad | 70500 (7.1) | 51300 (5.1) | 46600 (4.66) | 20100 (2.0) | 6890 (0.7) | 5060 (0.51) | 3340 (0.33) | 1570 (0.16) | 5.77 |
| 10-Kus | 69100 (6.9) | 36800 (3.7) | 48000 (4.80) | 21500 (2.2) | 7340 (0.7) | 4770 (0.48) | 3040 (0.30) | 1310 (0.13) | 4.38 |
| 11-Sava | 66300 (6.6) | 73600 (7.4) | 41200 (4.12) | 15200 (1.5) | 7000 (0.7) | 5440 (0.54) | 3220 (0.32) | 1810 (0.18) | 1.95 |
| 12-VelMor | 67600 (6.8) | 37300 (3.7) | 44700 (4.47) | 18100 (1.8) | 13000 (1.3) | 5900 (0.59) | 3110 (0.31) | 1560 (0.16) | 5.12 |
| 13-Pek | 68900 (6.9) | 22900 (2.3) | 30900 (3.09) | 14800 (1.5) | 17500 (1.8) | 5880 (0.59) | 2360 (0.24) | 3627 (0.36) | 2.49 |
| Average | 70877 (7.2) | 56562 (5.7) | 41585 (4.2) | 18215 (1.8) | 8573 (8.6) | 5466 (5.5) | 3580 (0.36) | 1477 (0.15) | 5.38 |
| SD | 12875 | 19293 | 6587 | 3040 | 3257 | 1174 | 777 | 330 | 2.16 |
| RSD | 18.2 | 34.1 | 15.8 | 16.7 | 38.0 | 21.5 | 21.7 | 22.3 | 40.1 |

**Table S1b**. Experimental (in mg kg^-1^) and statistic data of the quantified minor and trace elements in the River Danube’s sediments by INAA

| Sample  code | Concentration of minor and trace elements, mg kg^-1^ | | | | | | | | | | | | | | | | | |
| --- | --- | --- | --- | --- | --- | --- | --- | --- | --- | --- | --- | --- | --- | --- | --- | --- | --- | --- |
|  | Ba | Zn | Cr | Sr | V | Rb | Ni | Cu | Co | As | Sc | Th | Cs | Hf | Sb | U | W | Ta |
| 1-Rit | 420.0 | 226 | 109 | 131.0 | 90.2 | 95.9 | 47 | 54.6 | 15 | 11.5 | 12.1 | 11.0 | 6.3 | 4.7 | 2.0 | 2.1 | 1.8 | 0.9 |
| 2-SmedDS | 503.0 | 67 | 149 | 168.0 | 98.5 | 92.6 | 86 | 67.9 | 16 | 16.1 | 12.1 | 10.5 | 6.2 | 5.8 | 1.4 | 2.6 | 1.8 | 1.0 |
| 3-SmedRB | 368.0 | 62 | 153 | 158.0 | 77.0 | 75.6 | 87 | 40.4 | 14 | 4.2 | 10.7 | 7.8 | 5.1 | 4.3 | 1.4 | 2.7 | 1.3 | 0.7 |
| 4-Smed | 559.0 | 647 | 254 | 178.0 | 111.0 | 112.0 | 98 | 49.4 | 21 | 26.2 | 14.9 | 14.7 | 8.7 | 5.5 | 10.1 | 2.5 | 2.4 | 1.0 |
| 5-Ram | 505.0 | 371 | 193 | 174.0 | 135.0 | 127.0 | 113 | 41.1 | 24 | 21.1 | 16.7 | 13.9 | 10.2 | 4.9 | 3.4 | 2.4 | 2.4 | 1.0 |
| 6-VelGrad | 487.0 | 261 | 191 | 183.0 | 174.0 | 111.0 | 108 | 45.5 | 23 | 17.2 | 15.0 | 11.9 | 9.0 | 5.7 | 2.8 | 2.3 | 2.0 | 1.0 |
| 7-DoMil | 503.0 | 292 | 184 | 168.0 | 160.0 | 129.0 | 121 | 74.6 | 24 | 21.4 | 17.5 | 13.5 | 10.9 | 4.8 | 3.3 | 2.3 | 2.3 | 1.0 |
| 8-Tek | 535.0 | 299 | 185 | 167.0 | 136.0 | 132.0 | 110 | 78.7 | 25 | 19.0 | 17.3 | 13.4 | 10.8 | 5.1 | 3.5 | 2.4 | 2.3 | 1.0 |
| 9-Klad | 518.0 | 297 | 168 | 169.0 | 124.0 | 125.0 | 90 | 66.2 | 23 | 21.4 | 16.4 | 14.9 | 9.8 | 4.8 | 3.1 | 2.2 | 2.2 | 1.0 |
| 10-Kus | 545.0 | 329 | 175 | 162.0 | 119.0 | 130.0 | 107 | 55.9 | 24 | 19.9 | 17.3 | 15.4 | 10.3 | 4.8 | 4.4 | 2.3 | 2.2 | 1.0 |
| 11-Sava | 397.0 | 307 | 294 | 162.0 | 118.0 | 88.5 | 146 | 44.9 | 25 | 21.6 | 13.2 | 11.8 | 7.3 | 5.1 | 5.1 | 1.9 | 1.9 | 0.8 |
| 12-VelMor | 556.0 | 666 | 363 | 218.0 | 120.0 | 101.0 | 174 | 51.3 | 29 | 24.4 | 15.8 | 12.1 | 10.0 | 6.9 | 4.7 | 2.3 | 2.1 | 1.0 |
| 13-Pek | 459.0 | 425 | 75 | 219.0 | 114.0 | 58.6 | 24 | 512.6 | 15 | 11.0 | 12.9 | 7.7 | 2.3 | 4.6 | 5.2 | 1.4 | 1.9 | 0.7 |
| Average | 488.8 | 327 | 192 | 173.6 | 121.3 | 106.0 | 101 | 91 | 21 | 18 | 14.8 | 12.2 | 8.2 | 5.2 | 3.9 | 2.2 | 2.1 | 0.9 |
| SD | 61.2 | 179 | 76 | 23.4 | 26.3 | 23.1 | 38 | 127.3 | 5 | 6 | 2.3 | 2.5 | 2.6 | 0.7 | 2.2 | 0.3 | 0.3 | 0.1 |
| RSD | 12.5 | 55 | 39 | 13.5 | 21.7 | 21.8 | 38 | 140 | 22 | 34 | 15.7 | 20.3 | 31.8 | 13.2 | 57.9 | 14.7 | 14.7 | 13.3 |

**Table S1c.** Experimental (in mg kg^-1^) and statistic data of the quantified lanthanide elements in the River Danube’s sediments by INAA

| Sample  code | Concentration of lanthanide elements, mg kg^-1^ | | | | | | | | | | |
| --- | --- | --- | --- | --- | --- | --- | --- | --- | --- | --- | --- |
|  | La | Ce | Nd | Sm | Eu | Gd | Tb | Dy | Tm | Yb | ΣLn |
| 1-Rit | 31.6 | 56.6 | 37.3 | 3.8 | 1.5 | 3.9 | 0.7 | 4.1 | 0.3 | 2.5 | 142.31 |
| 2-SmedDS | 33.0 | 71.5 | 26.9 | 5.4 | 1.1 | 5.7 | 0.8 | 4.2 | 0.4 | 2.7 | 151.63 |
| 3-SmedRB | 24.4 | 55.5 | 16.2 | 4.2 | 1.2 | 4.1 | 0.7 | 2.9 | 0.5 | 2.6 | 112.23 |
| 4-Smed | 36.5 | 64.2 | 46.6 | 5.4 | 1.6 | 6.4 | 0.8 | 7.1 | 0.5 | 2.8 | 171.95 |
| 5-Ram | 36.1 | 63.1 | 39.7 | 3.6 | 1.9 | 4.7 | 0.8 | 6.5 | 0.4 | 3.0 | 160.03 |
| 6-VelGrad | 34.4 | 60.3 | 34.0 | 5.0 | 1.6 | 4.9 | 0.7 | 9.2 | 0.4 | 2.5 | 152.94 |
| 7-DoMil | 36.9 | 65.2 | 43.1 | 3.6 | 1.6 | 4.8 | 0.8 | 6.1 | 0.4 | 2.7 | 165.19 |
| 8-Tek | 36.8 | 65.2 | 35.2 | 4.9 | 1.8 | 4.6 | 0.9 | 5.7 | 0.4 | 3.3 | 158.60 |
| 9-Klad | 34.3 | 59.7 | 36.9 | 4.2 | 1.8 | 4.7 | 0.8 | 6.1 | 0.4 | 2.6 | 151.56 |
| 10-Kus | 35.7 | 63.7 | 37.9 | 3.2 | 1.9 | 4.2 | 0.8 | 5.5 | 0.4 | 2.8 | 156.11 |
| 11-Sava | 29.1 | 49.5 | 28.8 | 2.5 | 1.6 | 3.9 | 0.6 | 3.4 | 0.4 | 2.4 | 122.21 |
| 12-VelMor | 33.9 | 59.6 | 30.5 | 5.1 | 1.5 | 4.8 | 0.8 | 6.6 | 0.5 | 2.7 | 145.92 |
| 13-Pek | 21.7 | 37.5 | 31.9 | 3.5 | 1.5 | 4.0 | 0.6 | 5.9 | 0.3 | 2.2 | 108.97 |
| Average | 32.6 | 59.4 | 34.2 | 4.2 | 1.6 | 4.7 | 0.7 | 5.6 | 0.4 | 2.7 | 146.13 |
| SD | 4.8 | 8.5 | 7.7 | 0.9 | 0.2 | 0.7 | 0.1 | 1.7 | 0.1 | 0.3 | 20.6 |
| RSD | 14.8 | 14.3 | 22.6 | 21.6 | 15.5 | 15.5 | 12.5 | 29.6 | 16.9 | 10.5 | 14.1 |

**Figure S1.** Concentrations of the targeted lanthanides measured in the surface and deep sediments of the river Danube and its tributaries.


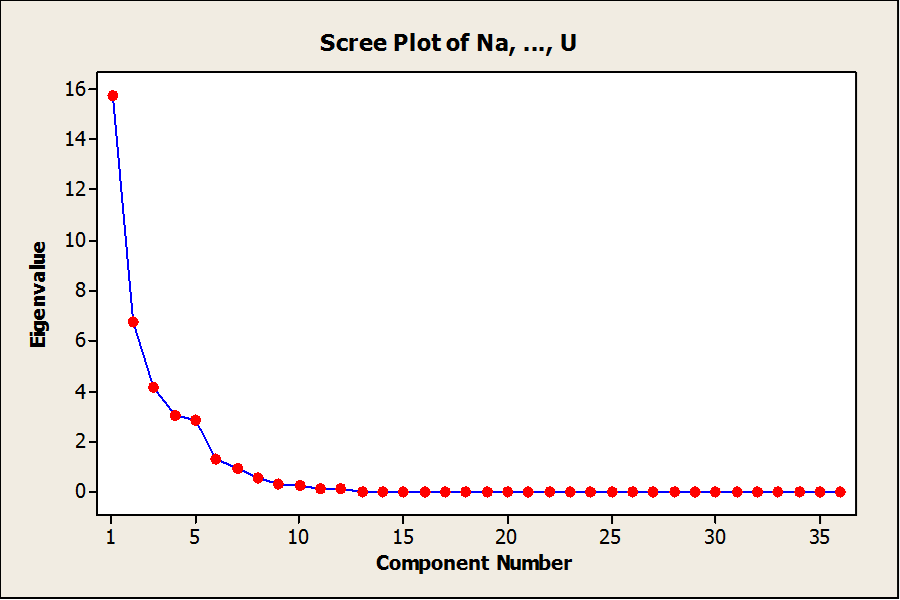


**Figure S2.** Component Eigen values for all studied elements.

**Table S2.** Eigen analysis of the Correlation Matrix

| Eigenvalue | 15.704 | 6.765 | 4.153 | 2.997 | 2.844 | 1.295 | 0.896 | 0.575 | 0.293 |
| --- | --- | --- | --- | --- | --- | --- | --- | --- | --- |
| Proportion | 0.436 | 0.188 | 0.115 | 0.083 | 0.079 | 0.036 | 0.025 | 0.016 | 0.008 |
| Cumulative | 0.436 | 0.624 | 0.740 | 0.823 | 0.902 | 0.938 | 0.963 | 0.979 | 0.987 |

**Table S3.** Rotated component matrix^a^

|  | Component | | | | | |
| --- | --- | --- | --- | --- | --- | --- |
|  | 1 | 2 | 3 | 4 | 5 | 6 |
| Rb | 0.937 |  |  |  |  |  |
| Th | 0.857 |  |  |  |  | 0.376 |
| Tb | 0.857 |  |  | 0.455 |  |  |
| La | 0.854 |  |  |  |  |  |
| Ta | 0.847 |  |  | 0.336 |  |  |
| K | 0.837 | 0.419 |  |  |  |  |
| Cs | 0.828 |  | 0.421 |  |  |  |
| Sc | 0.811 | 0.389 |  |  |  |  |
| Fe | 0.806 | 0.302 | 0.430 |  |  |  |
| Yb | 0.791 |  |  |  |  |  |
| Br | 0.786 |  |  |  | -0.323 | -0.305 |
| W | 0.758 | 0.329 |  |  |  | 0.457 |
| Ba | 0.695 |  |  | 0.431 | 0.445 |  |
| Ce | 0.690 |  |  | 0.566 | -0.352 |  |
| Eu | 0.690 |  |  | -0.495 |  |  |
| As | 0.558 |  | 0.469 |  |  | 0.527 |
| Ti |  | 0.963 |  |  |  |  |
| Al | 0.310 | 0.911 |  |  |  |  |
| V | 0.367 | 0.890 |  |  |  |  |
| Mg | 0.407 | 0.758 |  |  |  | 0.331 |
| Dy |  | 0.756 |  | 0.315 | 0.310 |  |
| Mn |  | 0.672 | 0.408 | -0.500 |  |  |
| Cr |  |  | 0.929 |  |  |  |
| Ni |  |  | 0.923 |  |  |  |
| Co | 0.531 | 0.362 | 0.719 |  |  |  |
| Tm | 0.336 |  | 0.628 | 0.545 |  |  |
| Sm |  |  |  | 0.934 |  |  |
| Gd |  |  |  | 0.841 |  | 0.379 |
| Hf |  |  | 0.607 | 0.610 |  |  |
| U | 0.384 |  |  | 0.563 | -0.538 |  |
| Ca |  |  |  |  | -0.898 |  |
| Sr |  |  | 0.327 |  | 0.815 |  |
| Na | -0.433 |  |  |  | 0.811 |  |
| Cu | -0.427 |  | -0.425 |  | 0.732 |  |
| Sb |  |  |  |  |  | 0.925 |
| Zn |  |  | 0.406 |  | 0.487 | 0.696 |
| Nd | 0.621 | 0.348 |  |  |  | 0.623 |
| ^a^Extraction Method: Principal Component Analysis, Rotation Method: Varimax with Kaiser Normalization.  ^a^Rotation converged in 16 iterations. | | | | | | |

Normalization.


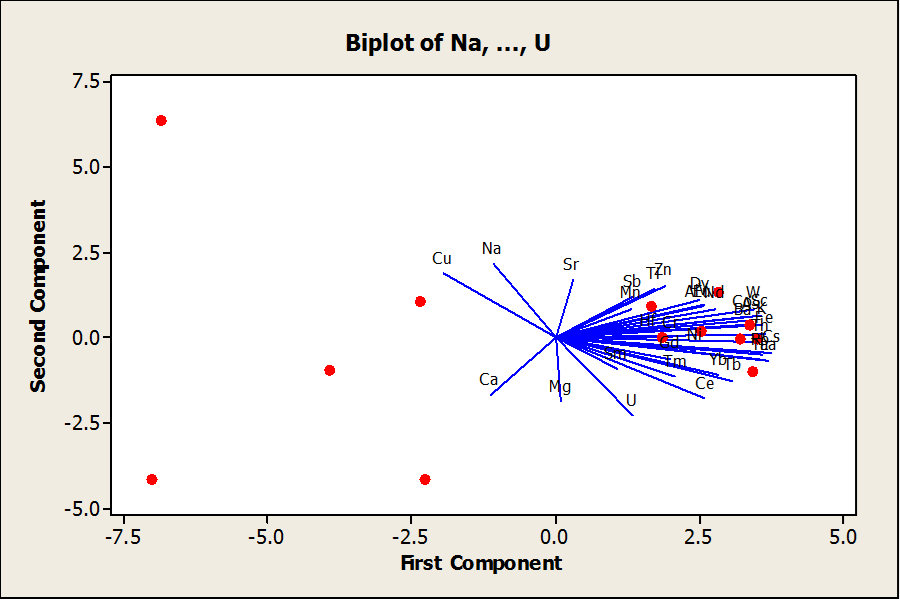


**Figure S3.** Score plot of PCA in analysis of total pool of concentrations of elements as vectors.

**Table S4.** Principal Component Analysis. (a) Initial Eigen Analysis; (b) Component matrix with 3 extracted components; (c) Component matrix after Varimax rotation

| **Total Variance Explained** | | | | | | | | | |
| --- | --- | --- | --- | --- | --- | --- | --- | --- | --- |
| Component | Initial Eigenvalues | | | Extraction Sums of Squared Loadings | | | Rotation Sums of Squared Loadings | | |
|  | Total | % of Variance | Cumulative % | Total | % of Variance | Cumulative % | Total | % of Variance | Cumulative % |
| 1 | 5.311 | 53.115 | 53.115 | 5.311 | 53.115 | 53.115 | 4.107 | 41.069 | 41.069 |
| 2 | 1.964 | 19.639 | 72.754 | 1.964 | 19.639 | 72.754 | 2.469 | 24.688 | 65.757 |
| 3 | 1.219 | 12.193 | 84.946 | 1.219 | 12.193 | 84.946 | 1.919 | 19.190 | 84.946 |
| 4 | 0.579 | 5.793 | 90.740 |  |  |  |  |  |  |
| 5 | 0.402 | 4.016 | 94.756 |  |  |  |  |  |  |
| 6 | 0.257 | 2.570 | 97.326 |  |  |  |  |  |  |
| 7 | 0.140 | 1.395 | 98.721 |  |  |  |  |  |  |
| 8 | 0.088 | 0.878 | 99.599 |  |  |  |  |  |  |
| 9 | 0.038 | 0.382 | 99.981 |  |  |  |  |  |  |
| 10 | 0.002 | 0.019 | 100.000 |  |  |  |  |  |  |


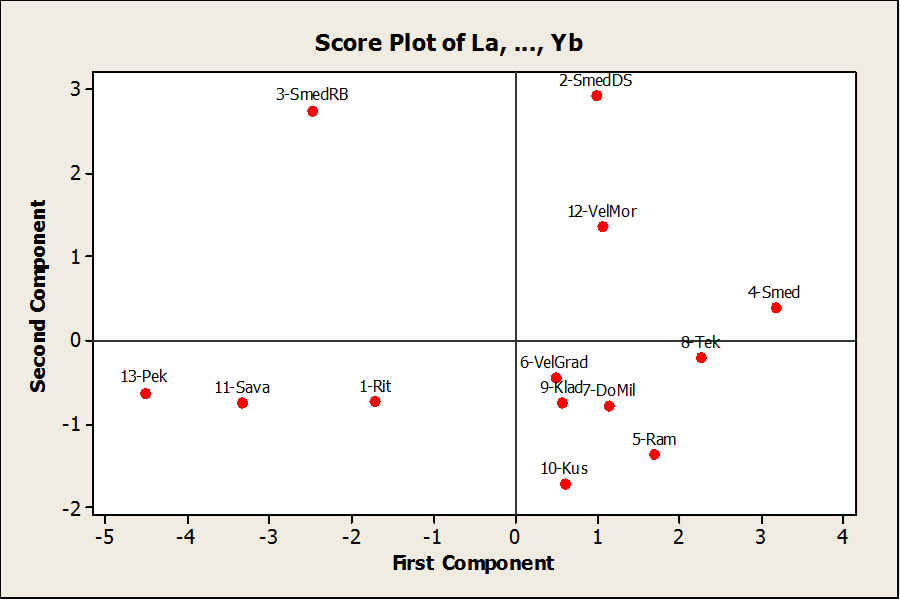


**Figure S4.** Principal component analysis of the studied lanthanides.
